# Prognostic significance of tumor size for primary invasive cutaneous melanoma: A population‐based study, 2004‐2016

**DOI:** 10.1002/cam4.3065

**Published:** 2020-05-05

**Authors:** Qiuying Ma, Huinan Suo, Li Zhu, Yue Qian, Xiaoyan Sun, Jun Xie, Qianru Li, Yangxue Fu, Jun Li, Juan Tao

**Affiliations:** ^1^ Department of Dermatology Union Hospital Tongji Medical College Huazhong University of Science and Technology (HUST) Wuhan China; ^2^ Hubei Engineering Research Center for Skin Repair and Theranostics Wuhan China; ^3^ Department of Dermatology Tongji Hospital Tongji Medical College Huazhong University of Science and Technology (HUST) Wuhan China; ^4^ Department of Dermatology Zhongnan Hospital Wuhan University Wuhan China; ^5^ Department of Dermatology The Central Hospital of Wuhan Tongji Medical College Huazhong University of Science and Technology (HUST) Wuhan China

**Keywords:** cutaneous melanoma, melanoma‐specific survival, staging, SEER, tumor size

## Abstract

**Background:**

This study aimed to assess the independent prognostic value of tumor size compared with other clinical and pathologic features of primary invasive cutaneous melanoma (CM).

**Methods:**

This study included 28,593 patients with primary invasive CM in Surveillance, Epidemiology, and End Results Program database diagnosed from 2004 through 2016. Tumor size was divided into five subgroups (≤6, 7‐12, 13‐30, 31‐42, and >42 mm). The primary endpoint was melanoma‐specific survival (MSS).

**Results:**

The relationship between tumor size and survival was piecewise. After adjusting for age, sex, primary site, histopathologic cell type, Breslow thickness, ulceration, mitotic rate, regional metastasis, and distant metastasis, the hazard ratio (HR) of MSS increased with increasing tumor size until a peak at 31‐42 mm (HRs, 1.33, 1.59, 2.41, respectively; all *P* < .0001), and then decreased when tumor size was larger than 42 mm using tumor size ≤ 6 mm as the reference (HR, 2.11; 95% confidence interval [CI], 1.84 −2.42; *P* < .0001). This pattern mostly remained after stratification by T subcategories from T1 to T4 in localized primary CM except that tumor size >42 mm subgroup had the shortest MSS in T4. In addition, tumor size with a cutoff value of 12 mm showed stronger prognostic value for MSS (HR, 2.32; 95% CI, 1.80‐2.98; *P* < .0001) than Breslow thickness and mitotic rate in primary CM with T1N0M0.

**Conclusions:**

Tumor size was an important independent prognostic factor for MSS in patients with primary invasive CM. Tumor size larger than 30 mm would provide additional and important prognostic information in each T subcategory of localized CM. Furthermore, tumor size with a cutoff value of 12 mm has great potential in improving the accuracy of melanoma T1 substaging.

## Graphical abstract

Tumor size was an important independent prognostic factor for MSS in patients with primary invasive CM. Tumor size larger than 30 mm would provide additional important prognostic information in each T subcategory of localized CM.Tumor size with a cutoff value of 12 mm has great potential in improving the accuracy of melanoma T1 substaging.

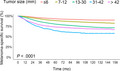

## 1 INTRODUCTION

Cutaneous melanoma (CM) continues to be a significant contributor to cancer morbidity and mortality.^1^, ^2^ Current assessment of survival outcomes of CM patients is based on the American Joint Committee on Cancer (AJCC) staging.^3^ The T category in the staging system is determined by Breslow thickness and ulceration. Breslow thickness was found to be of great value in assessing prognosis since 1970,^4^ and subsequent study validated that Breslow thickness with a cutoff point of 0.8 mm could separate high‐risk and low‐risk tumors.^5^ According to the 8th edition AJCC staging system, Breslow thickness ≥0.8 mm had a hazard ratio (HR) of 1.7 versus Breslow thickness <0.8 mm, but it was not statistically significant for melanoma‐specific survival (MSS) (*P* = .057).^3^ CM patients with localized tumors typically exhibit favorable prognosis, but recent studies have shown that patients with early stage CM contribute a substantial number of deaths from melanoma.^6^, ^7^ 31‐gene expression profile was reported to be capable to provide personalized prognostic information based on tumor biology.^8^, ^9^ Nonetheless, it could be time‐consuming and require specific equipment. Thus, it is still important to find useful clinicopathologic factors that may further stratify the patients with CM accurately, especially for those with early stage CM.

Recently, the tumor volume of CM has been reported to be prognostically superior to Breslow thickness in multivariate analysis,^10^, ^11^, ^12^ which implied that a multidimensional assessment could have more crucial prognostic implications than a single evaluation of Breslow thickness alone. Subsequently, Saldanha et al^13^ identified the independent prognostic value of tumor area calculated by tumor size and Breslow thickness for the 1239 cases (HR, 1.70), and it provided better stratification for MSS than Breslow thickness, which suggested that tumor size may have additional and important prognostic value for CM. However, these studies were based on complicated histological measurements and may be laborious. Meanwhile, tumor size has been included in the current staging of uveal melanoma.^14^To our knowledge, there were no comprehensive studies about the effects of tumor size on the prognosis of CM. Herein, detailed analyses about tumor size were conducted using data from the Surveillance, Epidemiology, and End Results (SEER) program database. We described the effects of tumor size on MSS in patients with primary invasive CM and further evaluated its prognostic effects on localized primary CM in each T subcategory.

## 2 METHODS

### 2.1 Data source

This project was not subject to institutional review board approval due to publicly accessible data. The right to conduct analysis was granted by the National Cancer Institute after signing the SEER data‐use agreement. SEERStat software was used to obtain and analyze data. SEER provides population‐based data on cancer incidence, treatment, and survival through 18 SEER cancer registries throughout the country for approximately 30% of the US population. Tumor size has been recorded for melanomas in the SEER database since 2004. For this reason, melanomas diagnosed from 2004 through 2016 were included in this study. Patients were identified based on the following order of inclusion criteria: invasive CM, primary by international rules, known precise tumor size, thickness, the status of ulceration, survival time, and status of regional and distant metastasis (Table 1). Finally, a total of 28,593 patients were included. About 21,361 patients with localized CM were characterized without either regional or distant metastasis (Table 1).

**TABLE 1:** Melanoma diagnosed, 2004‐2016

Melanoma categories	#
Total invasive cutaneous melanomas	254,569
Primary by international rules	232,731
Known precise tumor size	83,127
Known precise thickness	80,346
Known status of ulceration	79,195
Known survival time	79,194
Total samples with known status of both regional and distant metastasis	28,593
Total samples without both regional and distant metastasis	21,361

The data under the variables of “Collaborative Stage (CS) tumor size (2004‐2015)” and “Tumor Size Summary (2016+)” were combined for analysis. Surgery was performed for 98% of cases in this study. Tumor size was measured on pathology specimens by macroscopic examination and recorded in the pathology report. According to the annotation of the SEER database, tumor size was defined as the largest dimension of the primary tumor in millimeters and described the most accurate measurement of a solid primary tumor. Detailed records and coding rules could be found in Collaborative Staging Manual and Coding Instructions or available at: https://training.seer.cancer.gov/collaborative/system/tnm/t/size/. In addition, these cases were divided into thickness categories T1 through T4, consistent with the AJCC 8th edition staging system. Status of regional metastasis was determined by a comprehensive analysis of the variables below: “Regional nodes positive (1988+),” “CS lymph nodes (2004‐2015),” and “CS site‐specific factor 3 (2004+),” all of which recorded the status of regional disease in melanoma samples. “Regional nodes positive” records the exact number of positive regional lymph nodes (including sentinel lymph nodes) examined by the pathologist. “CS lymph nodes” obtained from regional lymphadenectomy records regional lymph node chain farthest from the primary site that is involved by tumor either clinically or pathologically (regional lymphadenectomy). “CS site‐specific factor 3” records the clinical status of lymph node metastasis (clinically detected metastasis). “Regional nodes positive (1988+),” “CS lymph nodes (2004‐2015),” and “CS site‐specific factor 3 (2004+)” were used to analyze the regional status of samples from 2004 to 2015. “Regional nodes positive (1988+)” and “CS site‐specific factor 3 (2004+)” were used to analyze the regional status of samples from 2016. According to the annotates of the SEER database, the contents under each variable were converted into negative, positive, and unknown status of regional disease. Then, the regional status of a single sample was determined as follows: The sample with negative results in all above three variables was defined as the sample without regional metastasis. The sample with positive results in any of the three variables was defined as the sample with regional metastasis. The remaining samples were unknown status of regional disease. Status of distant metastasis was determined by comprehensive analyses of “CS Mets at dx (2004‐2015),” “Mets at DX‐Distant LN (2016+),” and “Mets at DX‐Other (2016+),” all of which recorded the status of metastasis in distant organs or distant lymph nodes. The analysis process of distant metastasis was similar to the way that regional status analyzed.

### 2.2 Statistical analysis

Pearson Chi‐squared test for categorical variables and Kruskal‐Wallis rank‐sum test for non‐normally distributed continuous variables were performed to compare the distribution of melanoma by tumor size. The primary endpoint was MSS, which was defined as the time from diagnosis to a documented death due to melanoma. Kaplan‐Meier curves reflecting MSS were calculated using the log‐rank test. Cox proportional hazards models were used to identify significant predictors for MSS, and assumptions of proportionality were verified. All statistical analyses were performed with SPSS version 24.0 (SPSS Inc, Chicago, IL, USA) and R version 3.6.1 (http://www.r‐project.org). Statistical significance was set at two‐sided *P* < .05.

## 3 RESULTS

### 3.1 Patient and tumor characteristics

A total of 28,593 patients with primary invasive CM diagnosed from 2004 through 2016 were included in this study. Histopathologic subtypes of melanoma samples are shown in Table 2. After adjusting for age, sex, primary site, histopathologic cell type, Breslow thickness, ulceration, mitotic rate, regional metastasis, and distant metastasis in multivariate analysis, the pre‐calculated HR of tumor size as a continuous variable on MSS was 1.001 using Cox proportional hazards models. So, dividing tumor size into several subgroups was more appropriate for studying its effects on MSS. According to the ABCDE rule suggesting that the tumor size beyond 6 mm would provide a diagnostic clue for CM, tumor size was initially divided into 10 subgroups at 6‐mm intervals. HR of every tumor size subgroup on MSS was calculated using Cox proportional hazards models. Combining the distribution characteristics of melanomas by the 10 tumor size subgroups, tumor size subgroups with similar HRs were merged into a large subgroup for convenience. Thus, tumor size was finally divided into five subgroups, including tumor size ≤6 mm in 5893 cases (20.6%), tumor size 7‐12 mm in 9785 cases (34.2%), tumor size 13‐30 mm in 9323 cases (32.6%), tumor size 31‐42 mm in 1133 cases (4.0%), and tumor size >42 mm in 2459 cases (8.6%). The distribution of clinicopathologic characteristics of the primary invasive CM according to categories of tumor size is shown in Table 3. Median age, Breslow thickness, and the proportion of nodular and acral lentiginous melanoma, mitotic rate ≥1/mm^2^, ulceration, and regional metastasis increased with increasing tumor size until a peak at 31‐42 mm except that the peak of the proportion of male was at tumor size 13‐30 mm. When the tumor size was larger than 42 mm, the median value or percentage of the above factors decreased. The proportion of distant metastasis increased continuously until tumor size >42 mm (Table 3).

**TABLE 2:** The classification of histopathologic subtypes of 28,593 melanoma samples in this study

Histopathologic subtypes	Cases (%)
Malignant melanoma	11 456 (40.1)
Superficial spreading melanoma	8144 (28.5)
Nodular melanoma	5571 (19.5)
Acral lentiginous melanoma, malignant	703 (2.5)
Lentigo maligna melanoma	691 (2.4)
Spindle cell melanoma	684 (2.4)
Desmoplastic melanoma, malignant	623 (2.2)
Amelanotic melanoma	166 (0.6)
Mixed epithelioid and spindle cell melanoma	128 (0.4)
Epithelioid cell melanoma	125 (0.4)
Malignant melanoma, regressing	90 (0.3)
Balloon cell melanoma	12 (0.0)
Malignant melanoma in giant pigmented nevus	125 (0.4)
Malignant melanoma in junctional nevus	64 (0.2)
Blue nevus, malignant	10 (0.0)
Malignant melanoma in precancerous melanosis	1 (0.0)

**TABLE 3:** Clinicopathologic characteristics in 28,593 patients with primary invasive cutaneous melanoma by tumor size

Characteristics	Total n = 28,593 (100)	Tumor size				
		≤6 mm n = 5893 (20.6)	7‐12 mm n = 9785 (34.2)	13‐30 mm n = 9323 (32.6)	31‐42 mm n = 1133 (4.0)	>42 mm n = 2459 (8.6)
Age (y)	61 (50‐72)	59 (47‐69)	60 (49‐71)	64 (53‐74)	65 (54‐76)	62 (52‐72)
Tumor size (mm)	11 (7‐20)	5 (3‐6)	10 (8‐10)	18 (15‐22)	37 (35‐40)	80 (55‐142)
Thickness (mm)	1.5 (1.0‐3.2)	1.2 (0.8‐1.9)	1.4 (0.9‐2.6)	2.0 (1.1‐4.5)	3.1 (1.2‐7.0)	1.9 (1.0‐3.2)
Follow‐up (mo)	45 (19‐86)	57 (25‐94)	48 (21‐89)	40 (17‐77)	27 (11‐61)	41 (15‐92)
Sex						
Female	11 116 (38.9)	2634 (44.7)	3909 (39.9)	3252 (34.9)	416 (36.7)	905 (36.8)
Male	17 477 (61.1)	3259 (55.3)	5876 (60.1)	6071 (65.1)	717 (63.3)	1554 (63.2)
Primary site						
Limbs	13 698 (47.9)	2926 (49.7)	4812 (49.2)	4342 (46.6)	506 (44.7)	1112 (45.2)
Trunk	9253 (32.4)	1619 (27.5)	3128 (32.0)	3250 (34.9)	386 (34.1)	870 (35.4)
Head/neck	5566 (19.5)	1334 (22.6)	1829 (18.7)	1702 (18.3)	236 (20.8)	465 (18.9)
Overlapping	31 (0.1)	6 (0.1)	7 (0.1)	12 (0.1)	2 (0.2)	4 (0.2)
Skin	45 (0.2)	8 (0.1)	9 (0.1)	17 (0.2)	3 (0.3)	8 (0.3)
Histopathology						
Superficial	8144 (28.5)	1742 (29.6)	3031 (31.0)	2624 (28.1)	235 (20.7)	512 (20.8)
Nodular	5571 (19.5)	973 (16.5)	1771 (18.1)	1997 (21.4)	300 (26.5)	530 (21.6)
Lentigo	691 (2.4)	139 (2.4)	238 (2.4)	244 (2.6)	32 (2.8)	38 (1.5)
Acral	703 (2.5)	74 (1.3)	146 (1.5)	341 (3.7)	65 (5.7)	77 (3.1)
Others	13 484 (47.2)	2965 (50.3)	4599 (47)	4117 (44.2)	501 (44.2)	1302 (52.9)
Mitotic rate (/mm^2^)						
<1	2970 (10.4)	727 (12.3)	1139 (11.6)	888 (9.5)	78 (6.9)	138 (5.6)
≥1	12 609 (44.1)	2511 (42.6)	4368 (44.6)	4317 (46.3)	526 (46.4)	887 (36.1)
Unknown	13 014 (45.5)	2655 (45.1)	4278 (43.7)	4118 (44.2)	529 (46.7)	1434 (58.3)
Ulceration						
No	19 692 (68.9)	4886 (82.9)	7266 (74.3)	5597 (60.0)	511 (45.1)	1432 (58.2)
Yes	8901 (31.1)	1007 (17.1)	2519 (25.7)	3726 (40.0)	622 (54.9)	1027 (41.8)
						
No	21 445 (75.0)	4999 (84.8)	7830 (80.0)	6463 (69.3)	601 (53.0)	1552 (63.1)
Yes	7148 (25.0)	894 (15.2)	1955 (20.0)	2860 (30.7)	532 (47.0)	907 (36.9)
Distant metastasis						
No	27 845 (97.4)	5830 (98.9)	9663 (98.8)	9067 (97.3)	1040 (91.8)	2245 (91.3)
Yes	748 (2.6)	63 (1.1)	122 (1.2)	256 (2.7)	93 (8.2)	214 (8.7)

Notes

*P* values are based on Pearson Chi‐square test for categorical variables and Kruskal‐Wallis rank‐sum test for non‐normally distributed continuous variables; all *P *< .001.

### 3.2 Tumor size was an independent prognostic factor for MSS of patients with primary invasive CM

**FIGURE 1:**
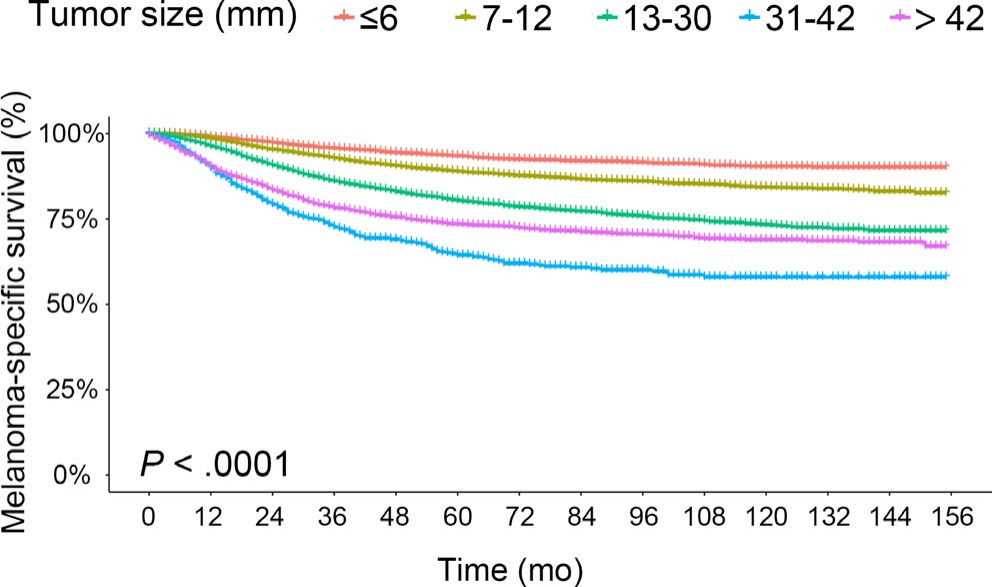
Survival analysis. Kaplan‐Meier curves are shown for predicted melanoma‐specific survival in five tumor size subgroups (≤6, 7‐12, 13‐30, 31‐42, and >42 mm) of patients with primary invasive cutaneous melanoma (n = 28,593). *P* value illustrates the difference between these curves, as calculated with the log‐rank test

There were 3649 patients (12.8%) who died because of melanoma. The median follow‐up was 45 months (interquartile range, 19‐86 months). The results of the Kaplan‐Meier survival analysis of MSS in the whole samples are displayed in Figure 1. After adjusting for age, sex, primary site, histopathologic cell type, Breslow thickness, ulceration, mitotic rate, regional metastasis, and distant metastasis in multivariate analysis, the HR of MSS increased with increasing tumor size until a peak at 31‐42 mm (HRs from 1.33 to 2.41; all *P* < .0001), after which increasing tumor size (>42 mm) was related to decreased HR (HR, 2.11; 95% confidence interval [CI], 1.84‐2.42; *P* < .0001), but was still greater than that of tumor size 13‐30 mm (Table 4). In addition, regional metastasis (HR, 3.70; 95% CI, 3.43‐3.98), distant metastasis (HR, 3.69; 95% CI, 3.32‐4.11), thickness class (HRs from 1.23 to 2.31), ulceration (HR, 1.81; 95% CI, 1.68‐1.95), and mitotic rate ≥1/mm^2^ (HR, 1.47; 95% CI, 1.19‐1.82) were associated with worse MSS as expected (all *P* < .0005). However, the prognostic value of age, primary site, and histopathology for MSS was significant but relatively small (HRs from 1.01 to 1.48) (Table 4).

**TABLE 4:** Association of tumor size with melanoma‐specific survival in whole samples with known status about regional and distant metastasis using Cox proportional hazards models (n = 28,593)

Variables	Univariate analysis		Multivariate analysis	
	HR (95% CI)	*P*	HR (95% CI)	*P*
Age (years)				
Continuous	1.02 (1.02‐1.02)	<.0001	1.01 (1.01‐1.02)	<.0001
Sex				
Female	Reference		Reference	
Male	1.68 (1.56‐1.80)	<.0001	1.28 (1.19‐1.38)	<.0001
Primary site				
Limbs	Reference		Reference	
Trunk	1.28 (1.19‐1.38)	<.0001	1.22 (1.13‐1.32)	<.0001
Head/neck	1.56 (1.43‐1.69)	<.0001	1.37 (1.26‐1.50)	<.0001
Histopathology				
Superficial	Reference		Reference	
Nodular	3.47 (3.15‐3.82)	<.0001	1.35 (1.21‐1.49)	<.0001
Lentigo	1.15 (0.87‐1.51)	.326	1.05 (0.80‐1.39)	.714
Acral	3.23 (2.70‐3.87)	<.0001	1.48 (1.23‐1.79)	<.0001
Tumor size (mm)				
≤6	Reference		Reference	
7‐12	1.70 (1.50‐1.92)	<.0001	1.33 (1.18‐1.51)	<.0001
13‐30	3.17 (2.82‐3.56)	<.0001	1.59 (1.41‐1.80)	<.0001
31‐42	6.42 (5.51‐7.49)	<.0001	2.41 (2.05‐2.82)	<.0001
>42	4.49 (3.93‐5.13)	<.0001	2.11 (1.84‐2.42)	<.0001
Mitotic rate (/mm^2^)				
<1	Reference		Reference	
≥1	3.50 (2.84‐4.30)	<.0001	1.47 (1.19‐1.82)	<.0005
Thickness (mm)				
≤1.0	Reference		Reference	
1.1‐2.0	1.49 (1.33‐1.67)	<.0001	1.23 (1.10‐1.38)	<.0005
2.1‐4.0	3.42 (3.07‐3.80)	<.0001	1.63 (1.46‐1.83)	<.0001
>4.0	7.94 (7.18‐8.78)	<.0001	2.31 (2.06‐2.59)	<.0001
Ulceration				
No	Reference		Reference	
Yes	4.18 (3.92‐4.47)	<.0001	1.81 (1.68‐1.95)	<.0001
Regional metastasis				
No	Reference		Reference	
Yes	6.62 (6.20‐7.08)	<.0001	3.70 (3.43‐3.98)	<.0001
Distant metastasis				
No	Reference		Reference	
Yes	13.37 (12.08‐14.79)	<.0001	3.69 (3.32‐4.11)	<.0001

Abbreviations: CI, confidence interval; HR, hazard ratio.

### 3.3 The prognostic value of tumor size in patients with localized primary CM in each T subcategory

**FIGURE 2:**
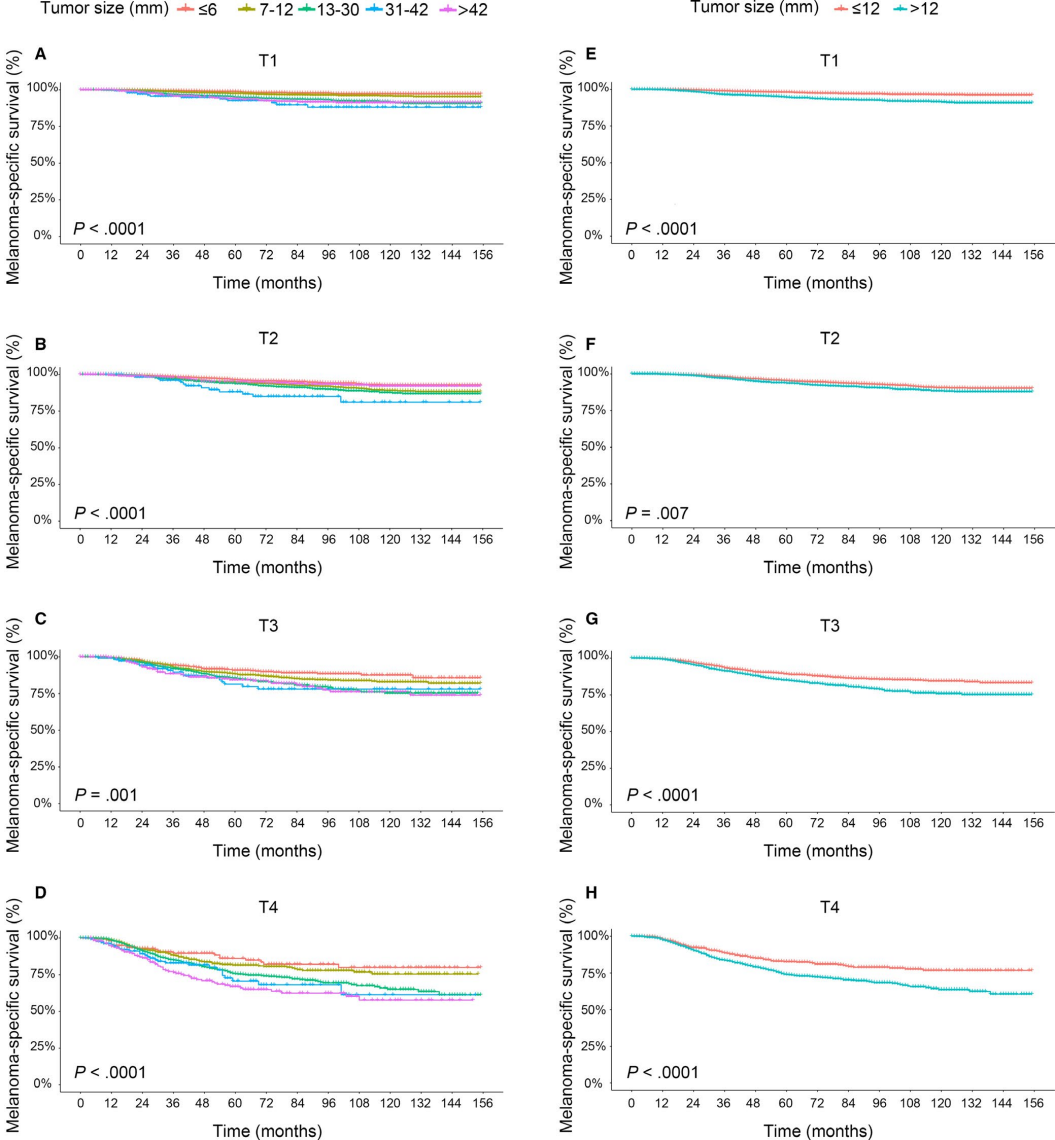
Survival analysis. Kaplan‐Meier curves are shown for melanoma‐specific survival in five tumor size subgroups (≤6, 7‐12, 13‐30, 31‐42, and >42 mm) (A, B, C, D) and two tumor size subgroups (≤12 and >12 mm) (E, F, G, H) of patients with localized primary cutaneous melanoma in each T category (n = 21,361). *P* value illustrates the difference between these curves, as calculated with the log‐rank test

We next investigated whether tumor size could further stratify the patients with localized primary CM in each T category. A total of 21,361 patients with localized primary CM were determined and used for analysis. The results of the Kaplan‐Meier survival analysis for MSS are shown in Figure 2A‐D. After adjusting for dichotomous ulceration and mitotic rate with a cutoff value of 1/mm^2^ in all T subcategories, and Breslow thickness with a cutoff value of 0.8 mm in T1, tumor size showed a significant and best prognostic value for MSS in T1 in all five subgroups (HRs from 1.63 to 4.10; all *P* < .05), and a certain degree of prognostic value for MSS in T2‐T4 (Table 5). In general, the HR of MSS increased with increasing tumor size until a peak at 31‐42 mm in all T subcategories, after which increasing tumor size (>42 mm) was related to decreased HR except that tumor size >42 mm subgroup had the shortest MSS in T4 (Table 5). Ulceration was associated with shorter MSS in every T subcategory (HRs from 1.85 to 4.78; all *P* < .0001). The effects of Breslow thickness and mitotic rate for MSS were not statistically significant in all T subcategories in multivariate analysis (Table 5).

**TABLE 5:** Association of tumor size with melanoma‐specific survival in each T category of localized primary cutaneous melanoma using Cox proportional hazards models (n = 21,361)

Variables	Univariate analysis		Multivariate analysis	
	HR (95% CI)	*P*	HR (95% CI)	*P*
T1 (n = 7943)				
Thickness ≥0.8 mm	0.80 (0.62‐1.03)	.078	0.88 (0.68‐1.13)	.300
Ulceration	5.23 (4.04‐6.77)	<.0001	4.78 (3.68‐6.19)	<.0001
Mitotic rate ≥1/mm^2^	2.07 (1.08‐3.97)	.029	1.78 (0.92‐3.41)	.085
Tumor size 7‐12 mm	1.63 (1.09‐2.44)	.017	1.63 (1.09‐2.44)	.017
Tumor size 13‐30 mm	3.29 (2.23‐4.85)	<.0001	3.03 (2.06‐4.47)	<.0001
Tumor size 3142 mm	4.87 (2.53‐9.38)	<.0001	4.10 (2.13‐7.92)	<.0001
Tumor size >42 mm	3.67 (2.30‐5.86)	<.0001	3.19 (2.00‐5.11)	<.0001
T2 (n = 7033)				
Ulceration	2.23 (1.78‐2.78)	<.0001	2.12 (1.70‐2.66)	<.0001
Mitotic rate ≥1/mm^2^	1.85 (1.07‐3.20)	.028	1.74 (1.01‐3.02)	.052
Tumor size 7‐12 mm	1.55 (1.16‐2.07)	.003	1.52 (1.14‐2.04)	.005
Tumor size 13‐30 mm	1.84 (1.37‐2.48)	<.0001	1.78 (1.32‐2.39)	<.0005
Tumor size 3142 mm	3.11 (1.72‐5.63)	<.0005	2.94 (1.62‐5.33)	<.0005
Tumor size >42 mm	1.20 (0.76‐1.90)	.433	1.19 (0.75‐1.88)	.464
T3 (n = 3925)				
Ulceration	1.89 (1.56‐2.30)	<.0001	1.85 (1.52‐2.25)	<.0001
Mitotic rate ≥1/mm^2^	1.71 (0.87‐3.36)	.122	1.52 (0.77‐2.99)	.230
Tumor size 7‐12 mm	1.32 (0.96‐1.81)	.088	1.24 (0.90‐1.70)	.189
Tumor size 13‐30 mm	1.76 (1.29‐2.41)	<.0005	1.63 (1.19‐2.24)	.002
Tumor size 31‐42 mm	2.00 (1.17‐3.42)	.011	1.89 (1.10‐3.23)	.020
Tumor size >42 mm	1.95 (1.29‐2.95)	.001	1.86 (1.23‐2.81)	.003
T4 (n = 2460)				
Ulceration	2.22 (1.80‐2.74)	<.0001	2.10 (1.69‐2.60)	<.0001
Mitotic rate ≥1/mm^2^	1.23 (0.57‐2.63)	.591	0.96 (0.45‐2.07)	.913
Tumor size 7‐12 mm	1.23 (0.77‐1.96)	.381	1.13 (0.71‐1.81)	.596
Tumor size 1330 mm	1.69 (1.09‐2.62)	.020	1.47 (0.94‐2.28)	.091
Tumor size 3142 mm	1.99 (1.14‐3.48)	.016	1.64 (0.93‐2.88)	.086
Tumor size >42 mm	2.47 (1.51‐4.02)	<.0005	1.97 (1.20‐3.23)	.007

Abbreviations: CI, confidence interval; HR, hazard ratio.

### 3.4 The prognostic value of dichotomous tumor size in thin melanomas

Considering the above results that the relatively similar MSS in tumor size subgroups no larger than 12 mm and the generally longer MSS for patients with size no larger than 12 mm compared with those larger than 12 mm, the prognostic value of tumor size with a cutoff value of 12 mm was further evaluated. The results of Kaplan‐Meier survival analysis for MSS are shown in Figure 2E‐H. With multivariate analysis, tumor size >12 mm subgroup showed shorter MSS in each T subcategory (HRs from 1.31 to 2.32; all *P* < .05) and best stratification ability in T1 (HR, 2.32; 95% CI, 1.80‐2.98; *P* < .0001) (Table 6) Ulceration was associated with shorter MSS in each T subcategory (HRs from 1.86 to 4.78; all *P* < .0001). The effects of Breslow thickness and mitotic rate for MSS were also not statistically significant in all T subcategories in multivariate analysis (Table 6).

**TABLE 6:** Effects of tumor size on melanoma‐specific survival in each T category of localized primary cutaneous melanoma (n = 21,361); the analysis of tumor size was performed with a cutoff value of 12 mm

Variables	Univariate analysis		Multivariate analysis	
	HR (95% CI)	*P*	HR (95% CI)	*P*
T1 (n = 7943)				
Thickness ≥0.8 mm	0.80 (0.62‐1.03)	.078	0.88 (0.68‐1.13)	.301
Ulceration	5.23 (4.04‐6.77)	<.0001	4.78 (3.69‐6.20)	<.0001
Mitoticrate ≥1/mm^2^	2.07 (1.08‐3.97)	.029	1.78 (0.92‐3.41)	.085
Tumor size >12 mm	2.56 (1.99‐3.28)	<.0001	2.32 (1.80‐2.98)	<.0001
T2 (n = 7033)				
Ulceration	2.23 (1.78‐2.78)	<.0001	2.16 (1.73‐2.71)	<.0001
Mitotic rate ≥1/mm^2^	1.85 (1.07‐3.20)	.028	1.70 (0.98‐2.95)	.057
Tumor size >12 mm	1.34 (1.09‐1.65)	.006	1.31 (1.07‐1.61)	.011
T3 (n = 3925)				
Ulceration	1.89 (1.56‐2.30)	<.0001	1.86 (1.53‐2.26)	<.0001
Mitotic rate ≥1/mm^2^	1.71 (0.87‐3.36)	.122	1.52 (0.77‐3.00)	.228
Tumor size >12 mm	1.49 (1.23‐1.81)	<.0001	1.45 (1.19‐1.76)	<.0005
T4 (n = 2460)				
Ulceration	2.22 (1.80‐2.74)	<.0001	2.13 (1.72‐2.63)	<.0001
Mitotic rate ≥1/mm^2^	1.23 (0.57‐2.63)	.591	0.97 (0.45‐2.08)	.938
Tumor size >12 mm	1.56 (1.25‐1.94)	<.0001	1.41 (1.13‐1.76)	.002

Abbreviations: CI, confidence interval; HR, hazard ratio.

Meanwhile, Spearman correlation analyses on tumor size with Breslow thickness and mitotic rate as continuous variables were performed. The correlation between tumor size and Breslow thickness was weak in whole samples (r, 0.234; *P* < .0001), also weak in T1‐T3 (r from 0.028 to 0.089; all *P* < .005), and slightly higher in T4 (r, 0.347; *P* < .0001) (Table 7). The correlation between tumor size and mitotic rate was still weak in whole samples (r, 0.217; *P* < .0001), also weak in T1, T2, and T4 (r from −0.050 to 0.090; all *P* < .005), not significant in T4 (*P* < .05) (Table 7) (all variables were non‐normally distributed).

**TABLE 7:** Association of tumor size with Breslow thickness and mitotic rate as continuous variables in each T category of primary invasive cutaneous melanoma using spearman correlation analysis

Variable	Breslow thickness		Mitotic rate	
	Spearman correlation	*P*	Spearman correlation	*P*
Tumor size	0.234	<.0001	0.217	<.0001
Tumor size in T1	0.053	<.0001	0.057	<.0001
Tumor size in T2	0.028	0.003	−0.050	<.0001
Tumor size in T3	0.089	<.0001	−0.009	0.551
Tumor size in T4	0.347	<.0001	0.090	<.0001

## 4 DISCUSSION

Tumor size was divided into five subgroups in this study (≤6, 7‐12, 13‐30, 31‐42, and >42 mm), which could provide essential and significant prognostic information of primary invasive CM. After adjusting for common prognostic factors such as Breslow thickness, ulceration, and mitotic rate, the HR of MSS increased with increasing tumor size until a peak at 31‐42 mm, and then decreased when tumor size was larger than 42 mm using tumor size ≤6 mm as the reference. The HRs of tumor size 31‐42 and >42 mm subgroups were higher than that of ulceration using tumors without ulceration as the reference. Interestingly, tumor size could further stratify the patients with localized primary CM in each T category. Most importantly, dichotomous tumor size with a cutoff value of 12 mm showed stronger prognostic value for MSS than Breslow thickness and mitotic rate in localized primary CM. It has great potential in improving the accuracy of current melanoma T1 subcategory. Accurate staging of T1 subcategory is also critical for the sentinel lymph node biopsy decision‐making.^15^

Because there were no other detailed reports about the effects of tumor size on the prognosis of CM, tumor size was initially stratified into 10 subgroups at 6‐mm intervals until tumor size >54 mm. The reason was that 6 mm was the diagnostic clue for CM suggested by ABCDE rule,^16^, ^17^ and the independent prognostic value of tumor size with a cutoff value of 12 mm was demonstrated in uveal melanoma.^18^ Then, the subgroups with similar HRs for MSS were merged. Five large subgroups were ultimately used in this study (≤6, 7‐12, 13‐30, 31‐42, and >42 mm). In this research, 20.6% of tumors were smaller than 6 mm, which was in accordance with the previous reports of 20%‐30% CM with tumor size ≤6 mm among primary invasive CM.^19^, ^20^, ^21^ Tumors with size 7‐12 mm accounted for the main part of all melanomas (34.2%). Tumors with size 31‐42 mm had a relatively small percentage (4.0%), but its highest HR for MSS compared with other tumor size subgroups possibly represented a turning point for the effects of tumor size on MSS. The HR of tumors size >42 mm subgroup was slightly lower than that of tumor size 31‐42 mm subgroup generally, but higher than that of tumor size 13‐30 mm subgroup. Together, five subgroups would make it easier to use.

There was only a small amount of literature describing the limited correlation between tumor size and Breslow thickness as this study revealed. Moreno‐Ramirez D et al^22^ observed that no statistical relationship existed between Breslow thickness and tumor size generally (r = 0.42, *P* > .01). Seidenari^23^ reported there was a weak relationship between tumor size and Breslow thickness in the trunk‐limbs group (r = 0.32, *P* <  .001). In addition, the limited correlation between tumor size and mitotic rate was also revealed. Nonetheless, the tumors in larger size subgroup had a higher proportion of malignant features, including thicker tumors, ulceration, mitotic rate ≥1/mm^2^, regional metastasis, and distant metastasis until the peak of 31‐42 mm. Patients with larger tumor size had concordant shorter MSS until tumor size 31‐42 mm, and the similar pattern mostly remained in each T subcategory of localized primary CM. Other multidimensional assessment of tumors also suggested that the larger tumor size subgroup with similar Breslow thickness would have worse prognosis.^10^, ^12^ For example, CM patients with tumor volume less than 140 mm^3^ had significantly higher 5‐year relapse‐free survival compared to those with larger tumors (98% vs 47%).^10^ Saldanha et al^12^ confirmed that the calculated tumor area had an HR of 1.70 for MSS. It could be that larger CM has undergone more cell divisions than smaller CM and may increase their chances of acquiring mutations that allow them to achieve selective growth advantage associated with poor prognosis.^24^ However, the prognosis of localized CM with tumor size >42 mm was not continuously worse than that of tumor size 31‐42 mm in T1‐T3 of localized primary CM as expected. Interestingly, the proportions of the aforementioned malignant features were correspondingly decreased in tumor size >42 mm subgroup compared with tumor size 31‐42 subgroup. We suppose that early stage CM with super large tumor size tends to grow radially rather than invasively, as Yu KD et al reported that those with 61‐ to 80‐mm tumors experienced significantly lower breast cancer‐specific mortality compared with those with 40‐ to 50‐mm tumors.^25^ In this result, the tumors larger than 42 mm had the shortest MSS in T4 of localized primary CM. One possible explanation is that a much thicker CM with a super larger tumor size may be related to a much heavier tumor burden, which may result in significantly shortest MSS. This hypothesis requires further investigations to confirm. In general, the simplicity of measurement and value for MSS of tumor size make it a priority for further study to assess its validity and to determine how it might be best translated into clinical application, compared with complex measurements of tumor volume or area.

Currently, Breslow thickness and ulceration are the two most important prognostic determinants for localized primary CM.^3^ Mitotic rate with a cutoff value of 1/mm^2^ was included as a T1 staging criterion of the 7th edition AJCC CM staging system.^26^ However, this study identified a higher HR of tumor size >12 mm than that of Breslow thickness and mitotic rate ≥1/mm^2^ (2.32 vs 0.88 and 1.78). Breslow thickness and mitotic rate were not statistically significant with multivariate analysis in this study, which was consistent with the results in the 8th edition AJCC staging system.^3^ It indicated that tumor size would have the potential to provide more accurate staging in thin melanomas with a cutoff value of 12 mm if verified in future studies. Besides, the HRs of tumor size 31‐42 and >42 mm subgroups would be higher than that of ulceration in some situations, although the percentage of tumor size >30 mm subgroup was only 12.6%. On the other hand, it should be noted that thickness and ulceration remained the two strongest predictors for MSS of primary localized CM in most instances, while tumor size is likely to offer additional important prognostic information.

This study has some limitations including the retrospective nature that could not exclude patient selection bias. Tumor size has been recorded in the SEER database since 2004. All available public data during 2004‐2016 were utilized in this study to achieve as high‐quality results as possible. Nevertheless, cases with known tumor size only account for 35.7% of all primary invasive CM during 2004‐2006, possibly because tumor size is not included in the required items of melanoma pathology reports based on the recommendations from the International Collaboration on Cancer Reporting.^27^ We may infer a selection bias for a greater‐than‐expected proportion of small tumor size melanoma among cases for which tumor size was not recorded. But the percentage of melanomas with small tumor size (≤6 mm) in the results was roughly consistent with the results reported by other investigators. Regarding this reason, the exclusion of these unknown tumor size cases from this analysis would have little effect. This study may bring to the attention of the clinical practitioners about recording tumor size in the pathology reports routinely and correctly. Accurate measurements are important for analyzing the role of tumor size in melanoma biology. Regrettably, few publications described the interobserver variability about tumor size. Additionally, the definitions of N and M subcategories were revised in the 8th edition AJCC staging system. Such updated staging is currently unavailable in the SEER database. Detailed substaging of regional and distant metastasis could not be obtained. Therefore, we distilled multiple data fields describing regional and distant metastasis, and performed strict comprehensive analyses for the status of the metastasis. Only melanomas that had no metastasis in all variables would be considered as localized CM, which ensured us to achieve the accuracy to the greatest extent possible. Besides, it is noted that Gemotty PA reported coding errors in SEER with respect to Breslow thickness in cases during 1988‐2010.^28^ As this study included data from 2004 through 2016, future studies are needed to confirm the results. Nevus‐associated melanoma is defined by the coexistence of nevus and melanoma features on histopathologic examination. Although melanocytic nevus has a very low likelihood of progressing to melanoma,^29^ a meta‐analysis reported that 29.1% of melanomas likely arose from a preexisting nevus and 70.9% de novo.^30^ But according to the classification of histopathologic subtypes of melanoma samples in this study, the percentage of nevus‐associated melanomas was less than 0.7%. Therefore, there would be little impact on the results. Nonetheless, the attempts to determine the proportion of tumors in nevus‐associated melanoma combining multiple immunohistochemical markers and genetic alterations are of great significance in future studies. Other endpoints, such as recurrence‐free survival, were also interesting. But we could not gather relevant information from the SEER database. This study may shed a light on the review of the prognostic value of tumor size with multiple endpoints.

This study is, to our knowledge, the first comprehensive description of tumor size on the prognosis of patients with CM. Tumor size has a significant prognostic value in whole samples and in each T subcategory of localized primary CM. Tumor size larger than 30 mm would provide additional important prognostic information for CM patients. Therefore, the recognition of quality and completeness of pathology reports about tumor size would improve patient care. Importantly, tumor size with a cutoff value of 12 mm showed a stronger prognostic value for MSS, which may assist in improving the accuracy of T1 substaging. These findings offered a useful and simple indicator for future prognostic models of primary invasive CM.

## CONFLICT OF INTERESTS

The authors declared no conflict of interest.

## AUTHOR CONTRIBUTIONS

Qiuying Ma: Conceptualization, data curation, visualization, and writing—original draft, review and, editing. Huinan Suo: Conceptualization and writing—review and editing. Li Zhu and Yue Qian: Methodology and data interpretation. Xiaoyan Sun and Jun Xie: Conceptualization, methodology, and writing—review and editing. Qianru Li and Yangxue Fu: Software, validation, formal analysis, data curation, and writing—review and editing. Jun Li and Juan Tao: Conceptualization, project administration, and writing—review and editing.

## Supporting information

Supplementary Material

## Data Availability

The data that support the findings of this study are available in The Surveillance, Epidemiology, and End Results (SEER) database (https://seer.cancer.gov/).

## References

[1] Siegel RL , Miller KD , Jemal A . Cancer statistics, 2019. CA Cancer J Clin. 2019;69(1):7‐34. 30620402 10.3322/caac.21551

[2] Shaikh WR , Dusza SW , Weinstock MA , Oliveria SA , Geller AC , Halpern AC . Melanoma thickness and survival trends in the United States, 1989 to 2009. J Natl Cancer Inst. 2016;108(1):djv294. 26563354 10.1093/jnci/djv294 PMC4857148

[3] Gershenwald JE , Scolyer RA , Hess KR , et al. Melanoma staging: evidence‐based changes in the American Joint Committee on Cancer eighth edition cancer staging manual. CA Cancer J Clin. 2017;67(6):472‐492. 29028110 10.3322/caac.21409 PMC5978683

[4] Breslow A . Thickness, cross‐sectional areas and depth of invasion in the prognosis of cutaneous melanoma. Ann Surg. 1970;172(5):902‐908. 5477666 10.1097/00000658-197011000-00017 PMC1397358

[5] Lo SN , Scolyer RA , Thompson JF . Long‐term survival of patients with thin (T1) cutaneous melanomas: a Breslow thickness cut point of 0.8 mm separates higher‐risk and lower‐risk tumors. Ann Surg Oncol. 2018;25(4):894‐902. 10.1245/s10434-017-6325-1 29330716

[6] Howlader N , Noone AM , Krapcho M , et al. SEER Cancer Statistics Review, 1975‐2014.. Bethesda, MD: National Cancer Institute, https://seer.cancer.gov/csr/1975_2014/, based on November 2016 SEER data submission, posted to the SEER web site, April 2017.

[7] Landow SM , Gjelsvik A , Weinstock MA . Mortality burden and prognosis of thin melanomas overall and by subcategory of thickness, SEER registry data, 1992–2013. J Am Acad Dermatol. 2017;76(2):258‐263. 27887797 10.1016/j.jaad.2016.10.018

[8] Gerami P , Cook RW , Wilkinson J , et al. Development of a prognostic genetic signature to predict the metastatic risk associated with cutaneous melanoma. Clin Cancer Res. 2015;21(1):175‐183. 25564571 10.1158/1078-0432.CCR-13-3316

[9] Gerami P , Cook RW , Russell MC , et al. Gene expression profiling for molecular staging of cutaneous melanoma in patients undergoing sentinel lymph node biopsy. J Am Acad Dermatol. 2015;72(5):780‐785.e3. 25748297 10.1016/j.jaad.2015.01.009

[10] Walton RG , Kim J , Velasco C , Swetter SM . Tumor volume: an adjunct prognostic factor in cutaneous melanoma. Cutis. 2014;94(5):226‐230. 25474450

[11] Voss B , Wilop S , Jonas S , et al. Tumor volume as a prognostic factor in resectable malignant melanoma. Dermatology. 2014;228(1):66‐70. 24335197 10.1159/000356121

[12] Friedman RJ , Rigel DS , Kopf AW , et al. Volume of malignant melanoma is superior to thickness as a prognostic indicator. Preliminary observation. Dermatol Clin. 1991;9(4):643‐648. 1934639

[13] Saldanha G , Yarrow J , Elsheikh S , O'Riordan M , Uraiby H , Bamford M . Development and initial validation of calculated tumor area as a prognostic tool in cutaneous malignant melanoma. JAMA Dermatol. 2019;155(8):890‐898. 10.1001/jamadermatol.2019.0621 PMC6596334 31241720

[14] Shields CL , Kaliki S , Furuta M , Fulco E , Alarcon C , Shields JA . American Joint Committee on Cancer Classification of Uveal Melanoma (Anatomic Stage) predicts prognosis in 7,731 patients: the 2013 zimmerman lecture. Ophthalmology. 2015;122(6):1180‐1186. 25813452 10.1016/j.ophtha.2015.01.026

[15] Swetter SM , Tsao H , Bichakjian CK , et al. Guidelines of care for the management of primary cutaneous melanoma. J Am Acad Dermatol. 2019;80(1):208‐250. 30392755 10.1016/j.jaad.2018.08.055

[16] Abbasi NR , Shaw HM , Rigel DS , et al. Early diagnosis of cutaneous melanoma: revisiting the ABCD criteria. JAMA. 2004;292(22):2771‐2776. 15585738 10.1001/jama.292.22.2771

[17] Friedman RJ , Rigel DS , Kopf AW . Early detection of malignant melanoma: the role of physician examination and self‐examination of the skin. CA Cancer J Clin. 1985;35(3):130‐151. 3921200 10.3322/canjclin.35.3.130

[18] Walter SD , Chao DL , Feuer W , Schiffman J , Char DH , Harbour JW . Prognostic implications of tumor diameter in association with gene expression profile for uveal melanoma. JAMA Ophthalmol. 2016;134(7):734‐740. 27123792 10.1001/jamaophthalmol.2016.0913 PMC4966166

[19] Crocetti E , Fancelli L , Caldarella A , Buzzoni C . Thickness and diameter in melanoma: is there a relation? Tumori. 2016;102(1):e1‐3. 26166224 10.5301/tj.5000369

[20] Maley A , Rhodes AR . Cutaneous melanoma: preoperative tumor diameter in a general dermatology outpatient setting. Dermatol Surg. 2014;40(4):446‐454. 24479783 10.1111/dsu.12454

[21] Abbasi NR , Yancovitz M , Gutkowicz‐Krusin D , et al. Utility of lesion diameter in the clinical diagnosis of cutaneous melanoma. Arch Dermatol. 2008;144(4):469‐474. 18427040 10.1001/archderm.144.4.469

[22] Moreno‐Ramirez D , Ojeda‐Vila T , Rios‐Martin JJ , Nieto‐Garcia A , Ferrandiz L . Association between tumor size and Breslow's thickness in malignant melanoma: a cross‐sectional, multicenter study. Melanoma Res. 2015;25(5):450‐452. 26237766 10.1097/CMR.0000000000000184

[23] Seidenari S , Fabiano A , Al Jalbout S , et al. Relationship between histological and computer‐based assessment of melanoma diameter and thickness in head versus trunk melanomas. Head Neck Oncol. 2013;5(3):32.

[24] Vogelstein B , Papadopoulos N , Velculescu VE , Zhou S , Diaz LA Jr , Kinzler KW . Cancer genome landscapes. Science. 2013;339(6127):1546‐1558. 23539594 10.1126/science.1235122 PMC3749880

[25] Yu K‐D , Jiang Y‐Z , Chen S , et al. Effect of large tumor size on cancer‐specific mortality in node‐negative breast cancer. Mayo Clin Proc. 2012;87(12):1171‐1180. 23218085 10.1016/j.mayocp.2012.07.023 PMC3541936

[26] Balch CM , Gershenwald JE , Soong S‐J , et al. Final version of 2009 AJCC melanoma staging and classification. J Clin Oncol. 2009;27(36):6199‐6206. 19917835 10.1200/JCO.2009.23.4799 PMC2793035

[27] Scolyer RA , Judge MJ , Evans A , et al. Data set for pathology reporting of cutaneous invasive melanoma: recommendations from the international collaboration on cancer reporting (ICCR). Am J Surg Pathol. 2013;37(12):1797‐1814. 24061524 10.1097/PAS.0b013e31829d7f35 PMC3864181

[28] Gimotty PA , Shore R , Lozon NL , et al. miscoding of melanoma thickness in SEER: research and clinical implications. J Invest Dermatol. 2016;136(11):2168‐2172. 27354265 10.1016/j.jid.2016.05.121 PMC5077675

[29] Shain AH , Bastian BC . From melanocytes to melanomas. Nat Rev Cancer. 2016;16(6):345‐358. 27125352 10.1038/nrc.2016.37

[30] Pampena R , Kyrgidis A , Lallas A , Moscarella E , Argenziano G , Longo C . A meta‐analysis of nevus‐associated melanoma: Prevalence and practical implications. J Am Acad Dermatol. 2017;77(5):938‐945.e934. 28864306 10.1016/j.jaad.2017.06.149

