# Supplementary material for: Prognostic significance of tumor size for primary invasive cutaneous melanoma: A population‐based study, 2004‐2016
Source: Cancer Med. 2020 May 5;9(13):4561–71. doi: 10.1002/cam4.3065 (PMC7333855; doi:10.1002/cam4.3065)
Supplement: Supplementary file 1 — Supplementary Material [file CAM4-9-4561-s001.docx]

**SUPPLEMENT TABLE 1.** Association of tumor size as a continuous variable with melanoma-specific survival in whole samples with known status about regional and distant metastasis using Cox proportional hazards models (n = 28,593).

|  |  | **Multivariate analysis** | |
| --- | --- | --- | --- |
| **Variables** |  | **HR (95% CI)** | ***P*** |
| Age (years) | Continuous | 1.01 (1.01-1.02) | <.0001 |
| Sex | Female | Reference |  |
|  | Male | 1.28 (1.19-1.38) | <.0001 |
| Primary site | Limbs | Reference |  |
|  | Trunk | 1.23 (1.14-1.34) | <.0001 |
|  | Head/ neck | 1.32 (1.21-1.44) | <.0001 |
| Histopathology | Superficial | Reference |  |
|  | Nodular | 1.32 (1.19-1.46) | <.0001 |
|  | Lentigo | 1.07 (0.81-1.41) | .631 |
|  | Acral | 1.52 (1.26-1.83) | <.0001 |
| **Tumor size (mm)** | **Continuous** | **1.001 (1.0003-1.001)** | **.0001** |
| Mitotic Rate (/mm^2^) | <1 | Reference |  |
|  | ≥1 | 1.50 (1.21-1.85) | <.0005 |
| Thickness (mm) | ≤1.0 | Reference |  |
|  | 1.1-2.0 | 1.22 (1.09-1.37) | <.0005 |
|  | 2.1-4.0 | 1.63 (1.46-1.83) | <.0001 |
|  | >4.0 | 2.50 (2.23-2.80) | <.0001 |
| Ulceration | No | Reference |  |
|  | Yes | 1.91 (1.77-2.05) | <.0001 |
| Regional metastasis | No | Reference |  |
|  | Yes | 3.83 (3.56-4.12) | <.0001 |
| Distant Metastasis | No | Reference |  |
|  | Yes | 3.91 (3.52-4.35) | <.0001 |

Abbreviations: HR, hazard ratio; CI, confidence interval.





**SUPPLEMENT FIGURE 1.** Distribution of melanomas. Tumor size was divided into 10 subgroups at 6 mm intervals (n = 28,593).

**SUPPLEMENT TABLE 2.** Association of tumor size with melanoma-specific survival in whole samples with known status about regional and distant metastasis using Cox proportional hazards models (n = 28,593); Tumor size was divided by 10 subgroups at 6 mm intervals.

|  |  | **Univariate analysis** | | **Multivariate analysis** | |
| --- | --- | --- | --- | --- | --- |
| **Variables** |  | **HR (95% CI)** | ***P*** | **HR (95% CI)** | ***P*** |
| Age (years) | Continuous | - |  | 1.01 (1.01-1.02) | <.0001 |
| Sex | Female | - |  | Reference |  |
|  | Male | - |  | 1.28 (1.19-1.38) | <.0001 |
| Primary site | Limbs | - |  | Reference |  |
|  | Trunk | - |  | 1.22 (1.13-1.32) | <.0001 |
|  | Head/ neck | - |  | 1.37 (1.26-1.50) | <.0001 |
| Histopathology | Superficial | - |  | Reference |  |
|  | Nodular | - |  | 1.35 (1.22-1.49) | <.0001 |
|  | Lentigo | - |  | 1.05 (0.80-1.39) | .728 |
|  | Acral | - |  | 1.48 (1.23-1.79) | <.0001 |
| **Tumor size (mm)** | **≤6** | **Reference** |  | **Reference** |  |
|  | **7-12** | **1.70 (1.50-1.92)** | **<.0001** | **1.33 (1.18-1.51)** | **<.0001** |
|  | **13-18** | **2.60 (2.28-2.96)** | **<.0001** | **1.48 (1.30-1.69)** | **<.0001** |
|  | **19-24** | **3.46 (3.01-3.98)** | **<.0001** | **1.65 (1.43-1.90)** | **<.0001** |
|  | **25-30** | **4.36 (3.77-5.03)** | **<.0001** | **1.76 (1.52-2.05)** | **<.0001** |
|  | **31-36** | **6.11 (5.02-7.44)** | **<.0001** | **2.23 (1.82-2.73)** | **<.0001** |
|  | **37-42** | **6.72 (5.59-8.08)** | **<.0001** | **2.59 (2.15-3.13)** | **<.0001** |
|  | **43-48** | **5.28 (3.98-7.01)** | **<.0001** | **1.73 (1.30-2.30)** | **<.0005** |
|  | **49-54** | **5.75 (4.50-7.36)** | **<.0001** | **2.27 (1.77-2.91)** | **<.0001** |
|  | **>54** | **4.24 (3.69-4.89)** | **<.0001** | **2.16 (1.87-2.49)** | **<.0001** |
| Mitotic Rate (/mm^2^) | <1 | - |  | Reference |  |
|  | ≥1 | - |  | 1.47 (1.19-1.82) | <.0005 |
| Thickness (mm) | ≤1.0 | - |  | Reference |  |
|  | 1.1-2.0 | - |  | 1.24 (1.10-1.39) | <.0005 |
|  | 2.1-4.0 | - |  | 1.63 (1.46-1.83) | <.0001 |
|  | >4.0 | - |  | 2.30 (2.05-2.58) | <.0001 |
| Ulceration | No | - |  | Reference |  |
|  | Yes | - |  | 1.81 (1.68-1.95) | <.0001 |
| Regional metastasis | No | - |  | Reference |  |
|  | Yes | - |  | 3.69 (3.42-3.97) | <.0001 |
| Distant metastasis | No | - |  | Reference |  |
|  | Yes | - |  | 3.65 (3.28-4.06) | <.0001 |

Abbreviations: HR, hazard ratio; CI, confidence interval.
